# Breastfeeding and the mother–child relationship: A case study of Ebonyi State University Teaching Hospital, Abakaliki

**DOI:** 10.4102/phcfm.v2i1.97

**Published:** 2010-04-09

**Authors:** Uche M. Okeh

**Affiliations:** 1 Industrial Mathematics and Applied Statistics, Ebonyi State University, Nigeria

## Abstract

**Background:**

The relationship between a mother and child is extremely important, especially with regard to breastfeeding habits. These affect the lives of children and mothers at an early stage and have become a source of concern for health workers and non-professionals alike.

**Objectives:**

This study was aimed at determining the relationships that exist between a mother and child and various breastfeeding habits.

**Method:**

The primary method of data collection was the design and use of a comprehensive questionnaire, which was distributed to women at the post-natal unit of the Gynaecology Department of Ebonyi State University Teaching Hospital in Abakaliki, Nigeria (EBSUTHAI). These women were civil servants, traders, students and housewives. A simple random sampling procedure of data collection was adopted in selecting the sample of 190 women. A chi-square method of analysis was used to test for independence of association. A 5% level of significance was considered.

**Results:**

At a 5% level of significance, a significant relationship existed between the category/occupation of mothers and the time intervals at which mothers breastfed their children (χ^2^_cal_= 20.53). Given the same level, exclusive breastfeeding was found to be dependent on a woman’s occupation (χ^2^_cal_= 8.49); however, at the same significance level, analysis showed that there was a significant relationship between a mother’s decision to feed her child breast milk as well as semi-solid food and those who chose to breastfeed exclusively (χ^2^_cal_ = 12.168). No significant relationship (χ^2^_cal_= 3.14) was found in determining whether children who are fed breast milk only are more intelligent than children who are fed semi-solid food as well.

**Conclusion:**

Mothers were expected to breastfeed their children at will because the time intervals at which they should breastfeed were not fixed. It seems that breastfeeding does not determine the intelligence of a child. Although it is generally recommended that mothers should practise exclusive breastfeeding, the findings of this study suggested that mothers should be equally recommended to alternate between feeding their children both semi-solid food and breast milk and breast milk exclusively, because a significant relationship exists between a mother’s decision to feed breast milk and semi-solid food as well as breastfeeding exclusively.

## INTRODUCTION

Breastfeeding is the feeding of an infant or young child with milk from a woman’s breasts. Babies have a sucking reflex that enables them to suck and swallow milk. With some exceptions, human breast milk is the best source of nourishment for human infants. However, some scholars disagree on how long to breastfeed to gain the greatest benefit and how much more risk is involved in using artificial formulas instead of breast milk. ^1^ While there are conflicting studies about the relative value of formula feeding, Riordan^2^ argues that formula feeding is inferior to breastfeeding for both full-term and premature infants. The Nigerian Federal Ministry of Health and international organisations, such as the World Health Organization (WHO), promote breastfeeding as the best method of feeding infants in their first year and beyond. There are numerous examples of research promoting exclusive breastfeeding - when an infant receives no other food or drink, including water, besides breast milk - and the use of human breast milk. ^3^ Regulatory authorities recognise the superiority of breastfeeding, but are also trying to make artificial feeding safer. ^4^ The American Academy of Pediatrics (AAP) also promotes exclusive breastfeeding and the use of human milk as the best method of feeding infants. ^5^

National and international guidelines recommend that all infants be breastfed exclusively for the first six months of life. According to the AAP, this is recommended so that the baby receives the ideal amount of nutrients necessary for optimal growth and development.^6^ Breastfeeding may continue with the addition of appropriate foods for two years or more. The WHO recommends - in those few health situations where infants cannot or should not be breastfed - the following alternatives: expressed milk from the infant’s own mother, breast milk from a healthy milk bank, or a breast milk substitute fed to the baby with a cup, which is a safer method than a feeding bottle because it is more nourished and pasteurised to make it germ free. The choice of one of these options is dependent on individual circumstances. Infants who are not breastfed, for whatever reason, should receive special attention from the health and social welfare system since they constitute a risk group. In other words, infants who are not breastfed are likely to be prone to infections.

Breastfeeding benefits mother and child both physically and psychologically. The promotion of breastfeeding and the use of human milk for infant feeding gives rise to a number of benefits, including health, nutritional, developmental, psychological, social, economic and environmental benefits.^3^ Early breast feeding of the child helps to combat the invasion of certain diseases and promotes the physical well being and sound brain development of the child. Exclusive breastfeeding has dramatically reduced infant deaths in developing countries by reducing cases of diarrhoea and infectious diseases.^7^ Breastfed babies also have a lower risk of sudden infant death syndrome (SIDS). ^8^ During breastfeeding, the nutrients, antibodies and beneficial hormones in the mother’s body are passed to her baby. Breast milk contains the amino acids cystine and taurine that are essential for the development of an infant’s brain and nervous system.^9^ Breast milk also has several anti-infective factors, including the anti-malarial factor para-amino benzoic acid. Sucking encourages the proper development of the infant’s teeth and speech organs and helps prevent obstruction. Breastfeeding is associated with a lower risk of the following ailments: allergies, asthma, breast cancer, diabetes, obesity and urinary tract infection.^10^

Breastfeeding also strengthens the bond between baby and mother, because breastfeeding releases the hormones oxytocin and prolactin, which relaxes the mother and makes her feel a sense of comfort and love for the baby during breastfeeding. As fat accumulated during pregnancy is used to produce milk, breastfeeding may help mothers lose weight. Exclusive breastfeeding can also delay the return of ovulation. Although some women may experience pain after breastfeeding as a result of a staphylococcal infection of the nipple, this can be easily managed with continued breastfeeding and treatment and should be of little concern for mother and child. Mothers who breastfeed their babies also have less risk of breast and endometrial cancer.^11^

Infants who are breastfed exclusively feed anywhere from six to 14 times a day, with newborns consuming between 30 mL and 90 mL of milk daily. After the age of four weeks, babies consume around 12 mL per feed. Each baby is different, but as he or she grows the amount of milk consumed generally increases. It is important to recognise the baby’s hunger signs because it is assumed that the baby knows how much milk they need and it is therefore advised to let the baby dictate the frequency and length of each feed. The supply of milk from the breast is determined by the number and length of these feeds or the amount of milk expressed, as well as other factors. A baby’s birth weight may also affect their feeding habits and mothers may be influenced by what they perceive the baby’s requirements to be. For example, a newborn who weighs less than they should for their gestational age, may lead a mother to believe that her child needs to feed more than if her child was larger; however, mothers should follow the demands of the baby rather than what they feel is necessary. While it can be difficult to measure how much food a breastfed baby consumes, babies normally feed to meet their own requirements. Babies who do not to eat enough may fail to thrive. If necessary, it is possible to estimate feeding from wet and soiled nappies (diapers) in the sense that eight wet cloth, or five to six wet disposable nappies and two to five soiled nappies per 24 hours suggest an acceptable estimate. However, stool frequency is a less accurate measure of adequate input because it is often normal for infants to go up to 10 days between stools. Babies can also be weighed before and after feeds, to determine the effect of their feeding habits on their growth.

## METHOD

The primary method of data collection was the administration of a questionnaire containing nine questions covering virtually every aspect of modern breastfeeding management. This questionnaire was distributed to various categories of women (civil servants, traders, students and housewives) at the post-natal care unit of Ebonyi State University Teaching Hospital, Abakaliki, Nigeria (EBSUTHAI) with the assistance of senior staff nurses. A sample size of 190 was considered using the simple random sampling technique. The chi-square test of independence of association was used for the analysis of the data generated. A 5% level of significance was considered.

## RESULTS

The analyses in this study were based on the data collected with regard to four hypotheses and were carried out on the basis of the observed and expected frequencies in the four contingency tables presented within this section. The figures in parentheses in Tables 1, 2 and 3 represent the expected frequency (Eij) for that specific test.

**TABLE 1:**
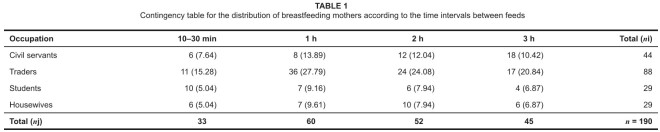
Contingency table for the distribution of breastfeeding mothers according to the time intervals between feeds

**TABLE 2:**
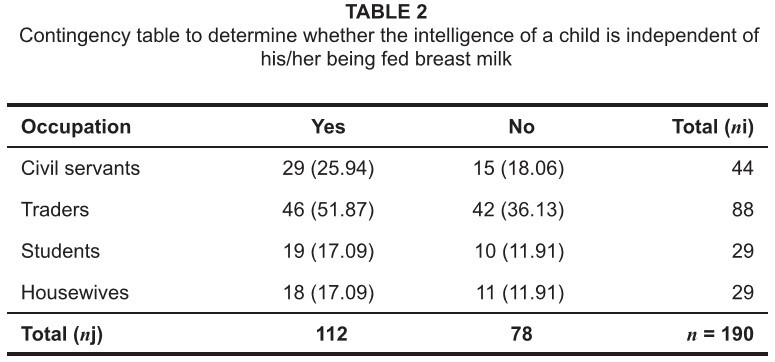
Contingency table to determine whether the intelligence of a child is independent of his/her being fed breast milk

**TABLE 3:**
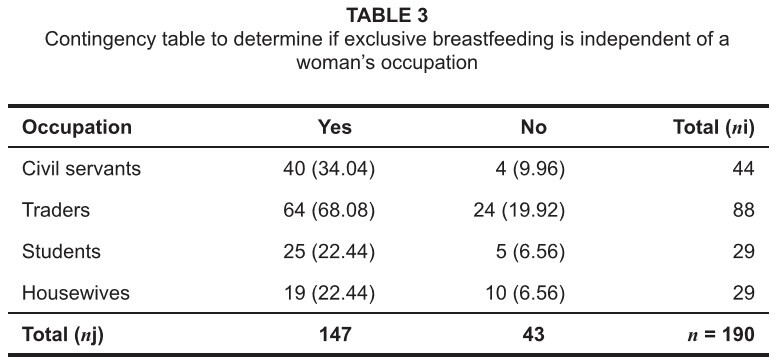
Contingency table to determine if exclusive breastfeeding is independent of a woman’s occupation

Table 1 shows the results of following hypothesis tests: 

- H_0_: The time intervals at which mothers breastfeed their children are independent of the category/occupation of mothers.
- H_1_: The time intervals at which mothers breastfeed their children are dependent on the category/occupation of mothers.

Results indicated that a significant relationship exists between the category/occupation of mothers and time intervals at which mothers breastfeed their children; at a 5% level of significance χ^2^_cal_= 20.53.

Table 2 shows the results of following hypothesis tests: 

- H_0_: Intelligence of a child is independent of being fed breast milk.
- H_1_: Intelligence of a child is dependent on being fed breast milk.

In the sampled population of 190, the number of women who agreed (based on responses to questions, see Table 2) that children fed only breast milk are as intelligent as children fed semi-solid food was higher (a total of 112). The null hypothesis was a test for independence of association. At a 5% significance level the chi-square calculated value is χ^2^_cal_ = 3.14. This means that there was no significant relationship between the intelligence of a child and the child feeding on breast milk.

Table 3 shows the results of following hypothesis tests: 

- H_0_: Exclusive breastfeeding is independent of occupation.
- H_1_: Exclusive breastfeeding is dependent on occupation. 

Analysis reflected that exclusive breastfeeding was dependent on occupation; χ^2^_cal_ = 8.49 at a 5% level of significance.

Table 4 shows the results of following hypothesis tests: 

- H_0_: A mother’s decision to feed both breast milk and semi-solid food is independent of exclusive breastfeeding.
- H_1_: A mother’s decision to feed both breast milk and semi-solid food is dependent on exclusive breastfeeding.

At a 5% level of significance χ^2^_cal_ = 12.168, which showed that there is a significant relationship between a mother’s decision to feed breast milk and semi-solid food to her child as well as breastfeeding exclusively.

**TABLE 4:**
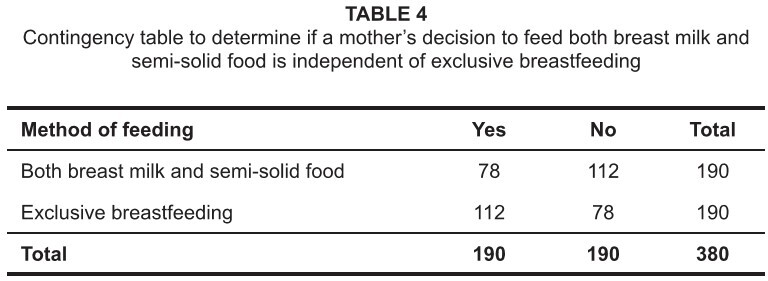
Contingency table to determine if a mother’s decision to feed both breast milk and semi-solid food is independent of exclusive breastfeeding

## DISCUSSION

Based on the findings of this study, it seems that exclusive breastfeeding is dependent on a mother’s occupation. Mothers who are students or traders, for example, do not have enough time, particularly at the early post-natal stage, to practise exclusive breastfeeding. Several studies also show that mothers who are employed, or anticipate returning to full-time employment, are less likely to breastfeed and when they do, they tend to feed their babies for a shorter length of time.^12,13^ This is contrary to the recommendation to breastfeed exclusively for at least a period of six months because human breast milk is the best source of nourishment for children and breastfed babies have a lower risk of various diseases. ^8^

These findings correspond with an earlier discovery that associations between breastfeeding and improved mother–child relations may, at least in part, reflect improvements in child cognitive functioning associated with breastfeeding.^14^ Studies on the nutritional and cognitive advantages associated with breastfeeding provide ample proof of the value of breastfeeding. There is also evidence of small, but consistently positive effects of breastfeeding on intellectual development. ^15,16^ This needs to be taken into consideration when preparing material that promotes breastfeeding.^14^ Specifically, women who choose not to breastfeed or who breastfeed for a shorter length of time tend to be younger, less educated, sole parents and poorer, and also reportedly have lower levels of parental nurturance.^17,18,19,20^ Based on the findings of this study, the time intervals at which mothers breastfeed their children is significant because a child requires regular breastfeeding for his or her early development.

## CONCLUSION

The results indicated that a child’s intelligence does not depend on whether he or she was fed only breast milk, because, at a 5% significance level, the chi-square calculated value was χ^2^_cal_ = 3.14, which confirmed that there was no significant relationship between the intelligence of a child and their feeding on breast milk. Mothers were expected to breastfeed their children at will since the time intervals at which they should breastfeed have not been fixed. Even though breastfeeding does not determine the intelligence of a child, early and regular breastfeeding should be encouraged as it is vital for the overall development of the child.
